# A Comprehensive Evaluation of the Readability of Online Healthcare Materials Regarding Distal Radius Fractures

**DOI:** 10.7759/cureus.18188

**Published:** 2021-09-22

**Authors:** Andrea Mc Carthy, Catherine Bossut

**Affiliations:** 1 Orthopaedics, St. James's University Hospital, Dublin, IRL; 2 Orthopaedics, St. James's Hospital, Dublin, IRL

**Keywords:** internet access, internet technologies, online medical education, accessibility, healthcare education, distal radius fractures, orthopaedics surgery

## Abstract

Introduction

Distal radius fractures (DRFs) are among the most common upper limb fractures reviewed in the emergency and orthopaedic departments. Approximately 40% of these fractures are unstable and require fixation to improve limb function. Confronted with an impending operation, many patients will access the internet, looking for information and reassurance. Previous studies have suggested that orthopaedic healthcare websites are beyond the comprehension of their target audience.

Objective

To assess the readability of healthcare websites regarding DRFs.

Methods

The terms *distal radius fracture, broken wrist *and *wrist fracture* were searched on Google and Bing. Of 101 websites initially considered, 52 unique websites underwent evaluation using readability software. Websites were assessed using two common methods for assessing readabilty; the Reading Grade Level (RGL) and the Flesch Reading Ease Score (FRES). In line with recommended guidelines and previous studies, an RGL of sixth grade and under and a FRES score above 65 was considered acceptable.

Results

The mean score for the FRES index was 56.67 (SD: ± 19.6), which resulted in the majority of pieces assessed being classified as ‘fairly difficult to read'. The mean RGL was 8.61 (SD: ± 2.86); 17.3% of the websites assessed fulfilled the criteria of having an RGL of six or less. One way T-tests comparing the FRES and RGL mean scores against the acceptable standards showed that they failed to meet the acceptable indexes (FRES: P<0.004; 95% CI: -13.8 to -2.8; RGL: P<0.0001; CI: 1.8-3.4). ANOVA testing showed no significant difference based on category (FRES: P=0.791; RGL: P=0.101).

Conclusion

The level of comprehension required for online healthcare education materials related to distal radius fractures exceeds the recommended guidelines. Improving the readability content of these websites would enhance the internet’s usability as an educational tool as well as improve patient post-operative outcomes.

## Introduction

Distal radius fractures (DRFs) are extremely common orthopaedic injuries, accounting for up to 18% of all fractures in the elderly age group and 25% of all upper limb fractured encountered in the emergency department [1,2]. The higher incidence rates of this fracture type in the elderly result in substantial issues with loss of independence and function as well as resultant increased health care costs [3]. Based on extensive research and clinical practice, orthopaedic surgeons have developed multiple approaches for the treatment of distal radius fractures, including both conservative and non-conservative options depending on patient needs and co-morbidities [4]. Surgical treatment is used to restore function and allow for early wrist mobilisation. However, like all surgeries, it must be acknowledged that this is not without risk [4].

Confronted not only with a sudden reduction in function and independence as well as an impending operation, many patients may become understandably overwhelmed and frightened. They may be confused by the information clinicians are providing but are too embarrassed to ask further questions or seek clarity. Instead, these patients and their families will peruse the internet as a ‘quasi-second opinion’ in an attempt to gain more understanding of their injury and treatment [5]. Considering that internet penetrance is due to reach approximately 97% by 2023, and that research shows that 90% of patients believe the internet to be a reliable source of health care education and information, it can therefore be concluded that it is of the utmost important for the information on the internet to be as inclusive and accessible if we are, as physicians and health advocates, to ensure adequate health literacy [3-7]. Previous research in this area has shown that this is often not the case, orthopaedic related information in particular presented on the internet has been shown to vary widely in terms of accuracy, quality and readability levels.

Health literacy is defined as the comprehension of basic health information to a level of competence that allows the patient to use the information provided to make decisions that improve their health [8]. Previous studies in this field have comprehensively shown that lower levels of health literacy are keenly associated with increased post-operative complications and reduced rehabilitation compliance [6,7,9-12]. Patients with poorer health literacy are also more likely to re-present to the hospital, have increased inpatient stay lengths, increased postoperative morbidity and mortality and lower post operative satisfaction [6,7,9-12]. All of these negative associated outcomes result in increased healthcare costs [13-15]. It can be thus, surmised that improving the readability of a text, the ease with which it is read and understood, is paramount to improving health literacy and positively impacting patient’s resilience in the fact of an impending surgery.

Previous health literacy guidelines have been published by the United States Departments of Health and Human Services (USDHSS) and by the National Institute of Health (NIH) [6,7,15-16]. According to these institutions, over 88% of Americans are unable to fully understand the information provided to them regarding their health [6,7]. In a bid to combat the negative outcomes and high costs that may be associated with this health ‘illiteracy’, the USDHSS recommends that all patient education materials be written at a reading grade level (RGL) of no higher than the sixth grade [6,7,15]. However, previous studies in the area have shown that healthcare educational websites are rarely adherent to this criterion [5-7,16-21].

Based on our literature search, we have found only one other paper which examined the readability of information on DRF [19] which demonstrated a readability standard beyond the comprehension of most adults. However, this study was conducted nearly a decade ago; within that time frame, many more people have gained access and knowledge of the internet and are now more comfortable with it. Furthermore, the internet is always rapidly updating and evolving with every possibility that newer more accessible healthcare websites may have been created since the previous review occurred. In light of these updates and the potential improvement that may have occurred over the last decade, the aims of this study will be two-fold. Firstly, we aim to evaluate the readability of healthcare information on the internet with regards to DRFs and our second aim is to determine if there has been an improvement over the last decade.

## Materials and methods

In July 2021, the terms *distal radius fracture, broken wrist* and *wrist fracture *were searched using the two most popular search engines (Google and Bing) and as per previous studies similar to this the first two pages of website hits from each search term were evaluated (n=101) [6,7,21]. This limitation was applied based on the evidence from previous studies which has demonstrated that the majority of people do not scroll beyond the first two pages of website hits when researching something and that most people only look at the first page of hits [6,7,19-21]. Table 1 encompasses the amount of hits returned for each search engine and each search term.

**Table 1:** Results from searches.

Search string	Returned results
Google & distal radius fracture	5,550,000
Google & wrist fracture	430,000,000
Google & broken wrist	684,000,000
Bing & distal radius fracture	686,000
Bing & wrist fracture	5,360,000
Bing & broken wrist	5,240,000

**Figure 1:**
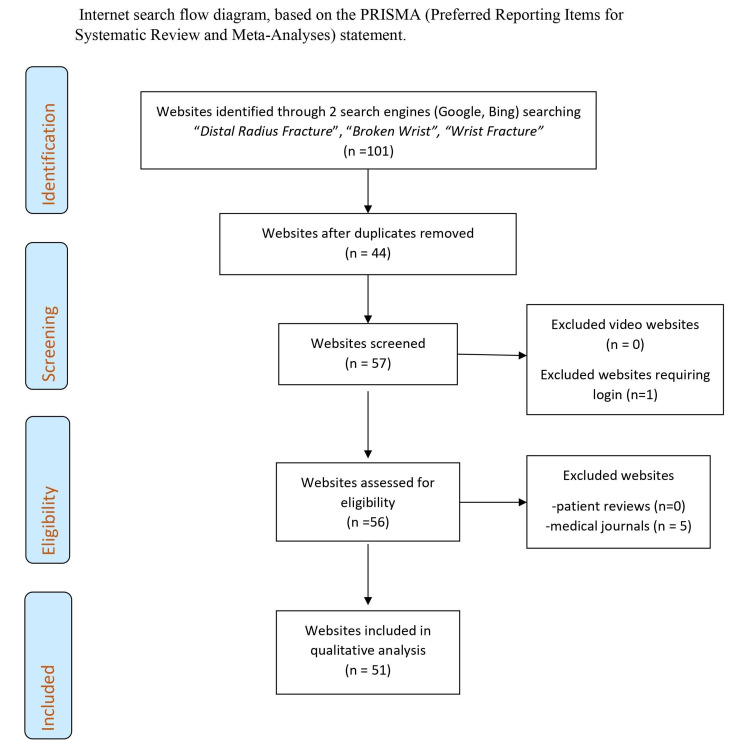
Website identification flow chart.

Duplicate websites were removed and medical journals, sites requiring logins or composed solely of videos were also excluded; previous authors had discerned that medical journals, with their extremely poor readability and accessibility indexes, required a significantly higher level of education to read and understand and would thus, be beyond the capability of the majority of the population [6,7,17-21]. Of the initial 101 websites, 52 unique web pages were identified as meeting the inclusion criteria and underwent further in-depth analysis [6,7,21]. A flow diagram showing a breakdown of this methodology is shown in Figure 1.

The websites were then categorised into physician, non-physician, commercial, media and news, social media and non-specified groupings [6,7,21]. 'Academic' refers to any website linked to a university while 'Physician' described any private website owned by a doctor [6,7,19-21]. 'Non-physician' websites referred to those created by other multidisciplinary team members such as physical therapists, occupational therapists or radiographers. 'Commercial' websites denoted websites which contained advertising or products to sell. 'Social media' is an umbrella category encompassing Facebook, Instagram and TikTok website hits to name a few, acknowledging their influence in the modern era. Sites that did not fall into any of the above categories were classed as 'Unspecified' [6,7,21]. Table 2 shows a list of included websites from both search engines (Google and Bing). This methodology has been used and validated in previous similar studies on the subject [6,7,19-21].

**Table 2:** List of included websites from the search engines.

Web browser	Included websites
Google	https://orthoinfo.aaos.org/en/diseases--conditions/distal-radius-fractures-broken-wrist/ https://en.wikipedia.org/wiki/Distal_radius_fracture https://www.hopkinsmedicine.org/health/conditions-and-diseases/distal-radius-fracture-wrist-fracture https://www.orthobullets.com/trauma/1027/distal-radius-fractures https://www.orthobullets.com/pediatrics/4014/distal-radius-fractures--pediatric https://www.hss.edu/conditions_distal-radius-fractures-of-the-wrist.asp https://teachmesurgery.com/orthopaedic/wrist-and-hand/distal-radius-fracture/ https://www.sports-health.com/sports-injuries/hand-and-wrist-injuries/treatment-distal-radius-fracture https://www.sports-health.com/sports-injuries/hand-and-wrist-injuries/broken-wrist-distal-radius-fracture https://www.mayoclinic.org/medical-professionals/orthopedic-surgery/news/distal-radius-fractures-in-older-versus-younger-patients/MAC-20429805 https://health.uconn.edu/orthopedics-sports-medicine/conditions-and-treatments/where-does-it-hurt/hand-and-wrist/distal-radius-fracture/ https://radiopaedia.org/articles/distal-radial-fracture https://www.physio-pedia.com/Distal_Radial_Fractures https://geekymedics.com/fractures-of-the-distal-radius-wrist-fractures/ https://rothmanortho.com/stories/blog/distal-radial-fracture-treatment-in-trenton https://www.msdmanuals.com/professional/injuries-poisoning/fractures/distal-radius-fractures https://www.assh.org/handcare/condition/wrist-fracture https://www.mayoclinic.org/diseases-conditions/broken-wrist/symptoms-causes/syc-20353169 https://www.nhs.uk/conditions/broken-arm-or-wrist/ https://healthcare.utah.edu/the-scope/shows.php?shows=0_oww13wsf https://www.guysandstthomas.nhs.uk/resources/patient-information/therapies/hand-therapy/advice-for-patients-with-a-fractured-wrist-in-a-plaster-cast.pdf https://www.sports-health.com/sports-injuries/hand-and-wrist-injuries/my-wrist-broken-or-sprained https://www.physioclinic.ie/conditions/broken-wrist/ https://www.yalemedicine.org/conditions/wrist-fracture/ https://www.esht.nhs.uk/wp-content/uploads/2017/06/0476.pdf https://www.stanfordchildrens.org/en/topic/default?id=broken-wrist-wrist-fracture-138-D1194 https://www.ouh.nhs.uk/patient-guide/leaflets/files/121210wrist.pdf https://myhealth.alberta.ca/Health/aftercareinformation/pages/conditions.aspx?hwid=uf7416 https://www.thephysiocompany.com/injury-or-condition/broken-wrist file:///C:/Users/Andrea/Downloads/Fractured%20wrist.pdf https://www.healthnavigator.org.nz/health-a-z/b/broken-wrist/ https://middlesexhealth.org/learning-center/diseases-and-conditions/broken-wrist https://www.guysandstthomas.nhs.uk/resources/patient-information/therapies/physiotherapy/general-advice-following-a-wrist-fracture-web.pdf https://www.ouh.nhs.uk/patient-guide/leaflets/files/121210wrist.pdf https://www.uofmhealth.org/conditions-treatments/cmc/hand-elbow-wrist/wrist-fractures https://www.healthline.com/health/colles-wrist-fracture
Bing	https://www.bssh.ac.uk/professionals/management_of_distal_radial_fractures.aspx https://en.wikipedia.org/wiki/Classification_of_distal_radius_fractures https://radiopaedia.org/articles/distal-radial-fracture?lang=gb https://www.sportsinjuryclinic.net/sport-injuries/wrist-pain/acute-wrist-injuries/broken-wrist https://www.epainassist.com/sports-injuries/wrist-injuries/broken-wrist https://www.drugs.com/cg/wrist-fracture-in-adults.html https://zangpt.com/broken-wrist-recover-faster/ https://www.sports-health.com/sports-injuries/hand-and-wrist-injuries/recovering-distal-radius-fracture https://patient.info/doctor/Wrist-Fractures https://bestpractice.bmj.com/topics/en-gb/392 https://www.thehealthexperts.co.uk/broken-fractured-bones/fractured-wrist/ https://sportsmedicine.mayoclinic.org/condition/hand-wrist-fractures/ https://pch.health.wa.gov.au/en/For-health-professionals/Emergency-Department-Guidelines/Fractures-Distal-forearm-or-wrist https://www.wikihow.com/Cope-With-a-Broken-Wrist https://theprehabguys.com/what-to-do-after-a-wrist-fracture/ https://www.bone-joint.com/different-types-of-wrist-fractures/

Once classified, the websites were uploaded into the online readability software (WEB FX) [6,7,22]. This software was then used to produce two readability scores for each of the websites; a Reading Grade Level (RGL) and a Flesch Reading Ease Score (FRES). The FRES score is defined as an index score used to determine the difficulty of understanding for any passage to be read and comprehended in English; this is done based on the number of syllables and the length of the sentences in each passage. It also accounts for the number of complex works in each passage. Complex words were defined as words with greater than three syllables or words with greater than 6 characters. Overly long sentences are defined as those with a word count greater than 22 words. The FRES score is the only readability testing metric where a higher score indicates an increased readability; a score of 65 or greater is considered to be acceptable [6,7,21-22]. A breakdown of the FRES scoring system and its interpretation is shown in Table 3.

**Table 3:** Breakdown of the Flesch Reading Ease Score system. A score of 65 or greater is concerned to be easily accessible to all reading levels [6,7].

Score	School level	Notes
100.00–90.00	5th grade	Very easy to read. Easily understood by an average 11-year-old student.
90.0–80.0	6th grade	Easy to read. Conversational English for consumers.
80.0–70.0	7th grade	Fairly easy to read.
70.0–60.0	8th & 9th grade	Plain English. Easily understood by 13- to 15-year-old students.
60.0–50.0	10th to 12th grade	Fairly difficult to read.
50.0–30.0	College	Difficult to read.
30.0–0.0	College graduate	Very difficult to read. Best understood by university graduates.

The reading grade level (RGL) was defined as cumulative score for the readability of a passage; this refers to the ease with which a person can read and understand a document or passage on the first pass [6,7,19]. As per previous studies, all reading grade levels are reported in terms of the US educational system and denote the number of years of formal schooling a person would need to have to easily read and comprehend the text [6,7,15,16]. As previously stated, it is recommended that healthcare-related materials be written at no more than a sixth-grade level of education [6,7,15,16]. To further determine accessibility, each website was assessed for translation services and if offered, how many translations were available.

Once this data had been determined, statistical analysis was undertaken; this was conducted using SPSS version 26 (SPSS, Chicago, IL) [23]. The level of statistical significance was set at 5%. ANOVA testing was performed between groups and if this achieved significance, Post-Hoc statistics were undertaken. A score of 65 or higher was determined to be acceptable for the FRES test; this acceptable standard was compared to the findings using a one-way t-test [6,7]. RGL was compared to the sixth grade standard using a one-way t-test [6,7].

## Results

**Figure 2:**
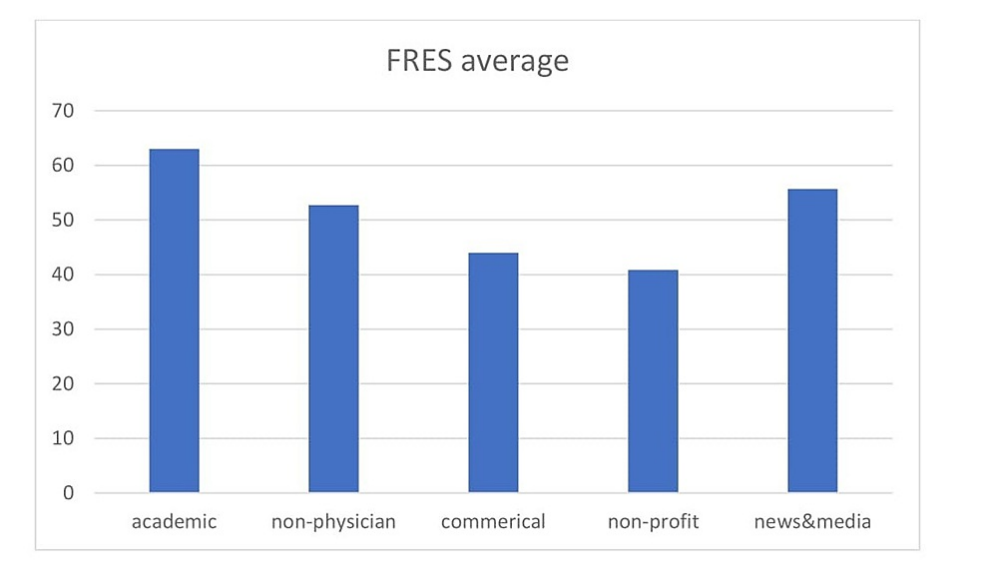
FRES mean scores by category. FRES: Flesch Reading Ease Score.

Of the initial 101 websites considered, 52 unique websites were evaluated using the readability tool [22]. This included 26 academic websites, 10 non-physician websites, three commercial, five non-profit and eight news and media websites. No physician, social media or unspecified websites were categorised. Of the 52 websites assessed, only 19 websites (36.5%) had a FRES score that met or exceeded the acceptable score standard of 65. The mean FRES index score was 56.67 (SD: ± 19.6), which resulted in the majority of websites assessed being classified as ‘fairly difficult to read (Figure 2). Eighteen of the reviewed websites (34.61%) had FRES scores between 30 and 50, suggesting a college level education would be required to be able to read and interpret them [6,7].

As shown in Figure 2, the highest-scoring category for the FRES index was academic websites; i.e. those linked to universities and teaching hospitals. A one-way t-test was performed comparing the FRES mean to the standard; this was significantly below the recognised acceptable index (P<0.004; 95% CI: -13.8 to -2.8). An ANOVA conducted showed no significant difference between FRES scores based on categories (P=0.791).

**Figure 3:**
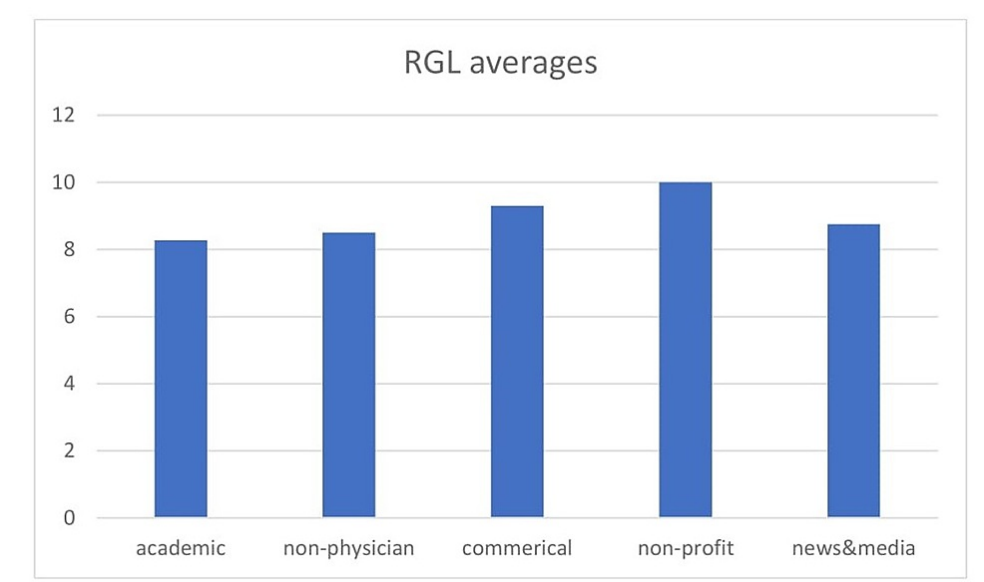
Mean RGL per category. RGL: Reading grade level.

In regards to the RGL, the mean score was 8.61 (SD: ± 2.86); this is the equivalent of an eighth-grade level of education [6,7]. Only 17.3% of the websites assessed fulfilled the acceptable criteria of having an RGL of six or less. Figure 3 demonstrates that the worst RGL scores were in the non-profit category while the best were the academic websites, followed by non-physician or allied health professionals websites. As per previous studies [6,7], one-way t-tests were conducted and demonstrated that these scores were significantly higher than the acceptable standard (P<0.0001; CI: 1.8-3.4) [6,7]. ANOVA testing showed no significant difference based on category (P=0.101).

## Discussion

Distal radius fractures are among the most common injuries encountered in orthopaedic practice, accounting for approximately 25% of all upper limb fractures reviewed in the emergency department [1-3]. Patients require access to highly comprehensible educational materials to ensure that they can give their full and explicit consent for the surgery [4-6,10-12,24]. Physicians must also be cognizant that many of their patients will have access to health care education materials of the internet which may provide invalid and inaccurate information to the patient, which can have a detrimental effect of patient outcomes and the patient-physician relationship if not addressed [6,7,12,24].

Similar to the trends exhibited in previous studies [11,16-21], this research showed that the majority of health education websites available which contained information about DRF are beyond the comprehension levels of the majority of the population. With both the FRES and RGL scoring significantly above the recommended standard, patients seeking additional information online run the risk of becoming confused and overwhelmed. This may affect their compliance with post-operative instructions and rehabilitation [6,7]. A lack of credible and accessible healthcare education material may also potentiate the risk of complications or lead to the patients developing cyberchondria [25].

It is considerably frustrating that despite guidelines provided by the NIH and USDHSS on the appropriate reading levels for these health education materials, the majority of the websites included in the study (82.7%) exceed this [13,14]. While this trend has improved from a previous study done in 2012 [20], which at the time of publication showed that 92% of studies exceeded the requirements, it can hardly be appraised a positive change when we consider that almost a decade has passed with only 10% improvement in the readability of the provided online health care education materials. When contemplated with the increasing amount of people whom have gained access to the internet during that timeframe and for forecast 97% penetrance of the internet by 2023, it can perhaps be theorised that no real progress has been made in improving the readability of health education materials overall.

Furthermore, it must be acknowledged that this study is not without limitations. Only the first two pages of each conducted search were analysed; while this was consistent with previous methodologies, it may also mean high-quality pages on later pages may have been excluded. The software used also determines the difficulty and readability of the websites based on the letters per word, syllables per word and number of words per paragraph [6,7,21,24]. This means that everyday words such as ‘disagreement’ may generate a higher RGL than words with fewer syllables and letters such as ‘physis’ which is a medical term and would be poorly understood by the general public [6,7,24].

## Conclusions

The information provided in healthcare websites online is beyond the scope of understanding of most potential patients and does not adhere to recommended guidelines. Despite a decade of guidance and advancements in accessibility on the internet, the improvement in the readability of the information surrounding distal radius fractures has been demonstrated to be a mediocre 10%. Steps should be taken to improve the readability of health care education materials based on the provided guidelines in a bid to improve post-operative satisfaction and compliance; this could be done by physicians creating their own accurate educational materials in line with the correct readability standards or providing additional education about the potential pitfalls of consulting "Dr Google" when preparing for an operation.
